# Generalizable and explainable deep learning for brain MRI: a multi-cohort evaluation of 3D architectures for age and sex prediction

**DOI:** 10.1186/s40708-026-00316-y

**Published:** 2026-07-06

**Authors:** Radhika Juglan, Marta Ligero, Zunamys I. Carrero, Asier Rabasco Meneghetti, Tim Lenz, Leo Misera, Gregory Patrick Veldhuizen, Paul Kuntke, Hagen H. Kitzler, Sven Nebelung, Daniel Truhn, Jakob Nikolas Kather

**Affiliations:** 1 Else Kroener Fresenius Center for Digital Health, Faculty of Medicine, TUD Dresden University of Technology, Dresden, Germany; 2 Department of Medicine I, Faculty of Medicine, TUD Dresden University of Technology, Dresden, Germany; 3 Medical Oncology, National Center for Tumor Diseases (NCT), University Hospital Heidelberg, Heidelberg, Germany; 4 Pathology & Data Analytics, Leeds Institute of Medical Research at St James’s, University of Leeds, Leeds, UK; 5 Department of Diagnostic and Interventional Radiology, University Hospital Aachen, Aachen, Germany; 6 Institute of Diagnostic and Interventional Neuroradiology, Faculty of Medicine and Carl Gustav Carus University Hospital, Technische Universität Dresden, Dresden, Germany; 7 Institute and Polyclinic for Diagnostic and Interventional Radiology, Faculty of Medicine and University Hospital Carl Gustav Carus Dresden, Technical University Dresden, Dresden, Germany

**Keywords:** Deep learning, Neuroimaging, External validation, Generalizability, MRI, Brain age

## Abstract

Deep learning (DL) methods increasingly outperform classical approaches in brain MRI analysis, yet their generalizability across independent imaging cohorts remains insufficiently evaluated. Because age and sex are fundamental neurobiological factors influencing brain structure and disease risk, this study systematically compares three three-dimensional architectures—Simple Fully Convolutional Network (SFCN), DenseNet121, and Swin Transformer—for age and sex prediction using T1-weighted MRI from four independent cohorts: UK Biobank (UKB, n = 47,390), Dallas Lifespan Brain Study (DLBS, n = 132), Parkinson’s Progression Markers Initiative (PPMI, n = 108 controls), and Information eXtraction from Images (IXI, n = 319). SFCN consistently demonstrated the most robust performance. For sex classification, it achieved an AUC of 1.00 [1.00–1.00] in the UKB internal test set and 0.85–0.91 across external cohorts. For age prediction, SFCN achieved a mean absolute error (MAE) of 2.66 years (r = 0.89) internally and 4.98–5.81 years (r = 0.55–0.70) externally. Pairwise DeLong and Wilcoxon tests with Bonferroni correction confirmed significantly better performance of SFCN compared with Swin Transformer in most cohorts (*p* < 0.017). No significant demographic subgroup biases were observed. Explainability analyses further showed task-specific and spatially consistent attention patterns across cohorts. These findings demonstrate that simpler convolutional architectures can generalize more reliably than more complex attention-based models in multi-cohort settings. The study highlights the importance of external validation and emphasizes potential trade-offs between model complexity, robustness, and interpretability for clinically relevant neuroimaging applications.

**Supplementary Information:**

The online version contains supplementary material available at 10.1186/s40708-026-00316-y.

## Introduction

Brain imaging analysis using Magnetic Resonance Imaging (MRI) has been key to identifying both anatomical and functional properties related to clinical factors, enabling earlier patient diagnosis and improved treatment [1–3]. Several studies have demonstrated the importance of structural T1-weighted MRI sequences in quantifying biomarkers for neurodegenerative diseases [4–18]. These biomarkers are quantifiable characteristics derived from T1-weighted imaging studies that indicate normal biological processes, pathologic processes, or responses to therapeutic intervention. Such biomarkers include various structural measurements, such as regional brain volumes, cortical thickness, white matter integrity, and morphometric features, which can be extracted to identify disease-specific patterns.

However, previous literature has shown that patients' demographic characteristics can significantly impact the development of diagnostic tools based on anatomical imaging. For example, sex-specific neurobiological changes have been associated with several neurodegenerative [19–23] and psychosocial diseases [24–27], while the effects of brain aging on disease progression have also been widely studied [28–30]. To address these factors, several studies have sought to predict biological variables, such as sex and age, directly from brain imaging studies [31–35] to better understand their neurobiology. However, these approaches require manual delineation to extract volumetric measurements. To overcome these limitations, Deep Learning (DL) has been explored as a means to develop end-to-end predictions from MRI without the need for manual annotations [36, 37].

DL has revolutionized medical image analysis by enabling automated and highly accurate predictions from complex imaging modalities, including T1-weighted brain MRI [36, 38–42]. In neuroimaging, 3-dimensional (3D) model architectures, such as 3D Convolutional Neural Networks (CNNs), have been particularly effective in capturing the volumetric structure of brain MRI data, providing superior performance over 2D models for tasks that require spatial context [40, 43–45]. These 3D models are crucial for applications such as brain age prediction, where spatial relationships across different brain regions play a key role [46, 47]. However, the emergence of newer model architectures, such as the Shifted Window (Swin) Transformer [48], which has demonstrated superior performance in 2D medical image tasks, particularly in the field of histopathology [49, 50], presents new opportunities for applying transformer-based models to 3D medical imaging. Swin Transformers, with their hierarchical structure and self-attention mechanisms, are well-suited for extracting both local and global features from images, yet their application to 3D neuroimaging tasks, such as the prediction of general clinical and biological properties remains largely unexplored.

Despite the advances in DL-based neuroimaging models for biological predictions [40, 45], several key challenges persist. Many models are trained and tested on the same cohort, raising concerns about their generalizability across diverse populations. For instance, several studies have relied on training and testing within the same cohort, casting doubt on the broader applicability of their findings [36, 41, 44, 45, 51–53]. Models often overfit specific cohort characteristics, resulting in performance degradation when applied to new datasets, as highlighted in previous research [39, 47].

The primary aim of this study is to systematically evaluate three different 3D deep learning architectures,—Simple Fully Connected Network (SFCN), Densenet121 and Swin Transformer—for predicting biological variables, specifically sex and age, directly from imaging data, while assessing their generalizability across external cohorts. Beyond predictive performance, we explore model assessment along three critical dimensions for clinical translation: (i) generalizability, through evaluation across independent external cohorts; (ii) bias characterization, by assessing the influence of subgroup-specific factors on model performance; and (iii) explainability, by applying gradient-based methods and volume-correlations to inspect model decision-making. Together, this work aims to expand model evaluation beyond isolated accuracy reporting toward a more trustworthy assessment framework, underscoring the importance of generalizability, explainability, and bias analysis for the future development of deep learning models in neuroimaging.

## Methods

### Data

**Fig. 1:**
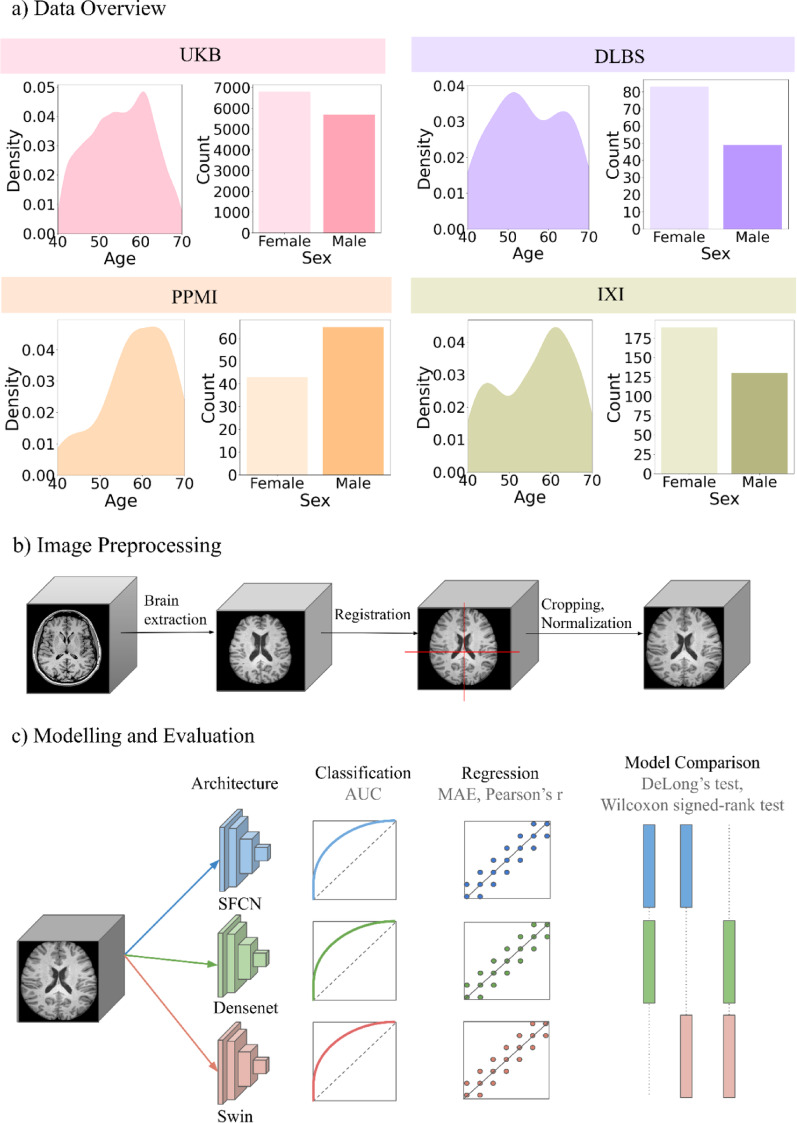
Study design. **a** Four data sets with T1-weighted structural brain MR imaging studies were used: UK Biobank (UKB), Dallas Lifespan Brain Study (DLBS), Parkinson's Progression Markers Initiative (PPMI), and Information eXtraction from Images dataset (IXI). **b** Images from all independent datasets went through the basic preprocessing steps of brain extraction, registration, cropping, and normalization before being fed to the 3D architectures. **c** Three different 3D architectures were trained for classification and regression tasks on the images from three of the four centers from UKB cohort, while images from the Newcastle center were held out as an independent test set. The best-trained models for each task were evaluated first on the Newcastle hold-out test set from UKB, and subsequently on three additional independent cohorts to assess generalizability. The tests were done using the metric of AUCs for sex classification and MAE and Pearson’s r for age prediction. Finally, the models were compared for statistical significance using the DeLongs test for the classification task and the Wilcoxon Signed-Rank test for the regression task

To evaluate model generalizability, we utilized four publicly available neuroimaging datasets: UK Biobank (UKB) [54], Dallas Lifespan Brain Study (DLBS), Parkinson’s Progression Markers Initiative (PPMI), and Information eXtraction from Images (IXI) (Fig. 1A, Table 1). The UKB cohort consists of high-resolution 3D T1-weighted brain MR images (Siemens Skyra 3T, MPRAGE sequence) with demographic labels for sex and age, collected across four imaging centers, with three centers used for training and one (Newcastle) held out for internal testing. The DLBS, PPMI, and IXI cohorts, used exclusively for external validation, also provide 3D T1-weighted MRI scans annotated with sex and age information. Further information on datasets can be found in Supplementary Table 1.

**Table 1:** Population characteristics

Cohort	Center	Type	Subjects	Sex distribution	Age distribution	% female	Age range
UKB	Cheadle	Train	34,918	18,215/16703	55.38 ± 7.60	52.17	[40, 71]
	Reading						
	Bristol						
	Newcastle	Test set 1	12,472	6784/5688	55.09 ± 7.45	54.40	[40, 71]
DLBS	n.a	Test set 2	132	83/49	55.08 ± 8.59	62.88	[40, 71]
PPMI	n.a	Test set 3	108	43/65	58.58 ± 7.77	39.81	[40, 71]
IXI	n.a	Test set 4	319	189/130	56.18 ± 8.61	59.25	[40, 71]

Demographic characteristics of the study cohorts. The datasets include UK Biobank (UKB) with data from multiple centers (training set: n = 34,918; test set 1; n = 12,472), and three external validation cohorts: Dallas Lifespan Brain Study (DLBS, test set 2; n = 132), Parkinson's Progression Markers Initiative (PPMI, test set 3; n = 108), and Information eXtraction from Images (IXI, test set 4; n = 319). The sex distribution is shown as female/male counts, with age presented as mean ± standard deviation. All cohorts span a common age range of 40–70 years. Center indicates specific data collection site; Type specifies dataset partition; Subjects shows total participant count; Sex distribution presents female/male numbers; Age distribution shows mean ± standard deviation; % female indicates percentage of female participants; Age range shows the minimum and maximum age in years

### Image preprocessing

To standardize input data across model training and testing, preprocessing was applied to DLBS, PPMI, and IXI, as outlined in Fig. 1B. UKB images were preprocessed following standard protocols [55]. To ensure spatial consistency across images, all brain MRI scans were registered to the Montreal Neurological Institute (MNI) space using the resseg-mni library [56, 57]. Brain extraction was performed using HD-BET [58] to remove non-brain structures and prevent models from learning confounding features. 3D Images were then intensity normalized (zero mean, unit variance) at the volume-level using TorchIO’s ZNormalization technique, followed by center-cropping to a standardized size of (180, 180, 180) voxels [59].

### Model architectures

We evaluated three existing 3D neural network architectures: Simple Fully Connected Network (SFCN) [37], Monai’s implementation [60] of DenseNet121 [61], and Shifted Window (Swin) Transformer [48], as shown in Fig. 1C. SFCN, the state-of-the-art model for brain age prediction, consists of a series of 3D convolutional layers followed by batch normalization and ReLU activations, concluding with global average pooling and fully connected layers for outputting a single age prediction or binary sex classification logits. Monai’s Densenet121 is a densely connected convolutional neural network that extends connectivity across three dimensions, enhancing feature reuse by allowing each layer to receive inputs from all preceding layers. This architecture facilitates efficient gradient flow through volumetric dense blocks, improving information propagation across the network. Lastly, Monai’s 3D Swin Transformer is a hierarchical transformer model that applies shifted window-based self-attention to 3D patches, enabling scalable and computationally efficient processing of volumetric data while capturing both local and global spatial dependencies across multiple resolutions.

### Training procedures

Models were trained on UKB using a training-validation split (2:1) with a fixed random seed (42). Hyperparameters were tuned on the validation set, and the final model performance was evaluated on the held-out test sets from different cohorts. Binary Cross-Entropy was employed as the loss function for the sex classification, while Mean Absolute Error (MAE) was utilized for regression (age prediction). A batch size of 4 and a learning rate of 10e−05 were fixed across all models. Early stopping was applied during training, halting the process if the validation loss failed to improve for 10 consecutive epochs, thereby reducing the risk of overfitting and selecting the last best model.

### Bias correction

To reduce systematic bias in age predictions, we applied a linear regression-based bias correction method [62]. For each model, predicted age values were regressed against their corresponding true age labels using the training dataset only. A linear regression model was fitted to these data points to establish a relationship between the predicted and actual ages. The resulting regression coefficients were then applied to the independent test sets, producing bias-corrected age estimates. After correction, MAE was calculated to quantify predictive accuracy, and Pearson's correlation coefficient (r) was computed to evaluate the linear relationship between the corrected predictions and true labels.

### Statistical methods

Model performance was evaluated using appropriate task-specific metrics. Area Under the Receiver Operating Characteristic Curve (AUC) was used to assess sex classification performance, while MAE and Pearson’s r were used to evaluate age prediction accuracy and linear association. To compare model performance in each cohort, statistical significance was assessed using the pairwise DeLong tests for AUC comparisons in classification tasks and the pairwise Wilcoxon signed-rank tests for paired differences in age prediction tasks followed by Bonferroni correction of alpha.

### Subgroup analysis

Following model evaluation, we conducted a subgroup analysis to assess demographic-related biases in the best-performing model. The test data was stratified by age groups (for sex classification) and sex classes (for age prediction) to evaluate potential performance disparities. This analysis provided insights into any demographic distribution-based biases or limitations in model generalizability.

### Explainability

To better understand the spatial focus of the models, we applied two complementary explainability approaches. First, Gradient x Input (I x G) attribution maps were generated to visualize the regions most responsible for driving the model’s predictions for age and sex. For each subject, the gradient of the predicted output with respect to the input image was computed and multiplied element-wise by the input voxel intensities, highlighting regions whose intensity jointly contributed to the model’s decision [63]. Heatmaps from all subjects in each cohort were averaged and overlaid onto a single representative brain image taken from each cohort which are visualized as 3D surface views using Mango image processing software (ric.uthscsa.edu/mango).

Second, we conducted a volume correlation analysis by correlating model prediction scores and ground truth (GT) labels with regional gray matter volumes from 139 Image-Derived Phenotypes (IDP) provided by UKBiobank [55]. For better visualizations, these 139 IDPs were grouped into nine broader anatomical regions as done in [64] using Freesurfer lobesStrict segmentation into frontal lobe, parietal lobe, occipital lobe, temporal lobe, limbic (or cingulate) lobe, insular lobe, subcortical structures, cerebellum, and brain stem. This analysis allowed us to quantify associations between model outputs and regional brain morphology, offering insights into task-specific anatomical relevance.

## Results

### Model training and population characteristics

The models were trained on 34,918 participants from the UKBiobank (UKB) aged 40–71 years, with a share of 52.2% female participants. To assess cross-center generalizability, we tested the models on unseen images from a held-out center within UKB, comprising 12,472 participants aged 40–71 years and 54.4% female participants (Test Set 1, “internal test set”). To evaluate cross-cohort generalizability, we tested the models on three independent datasets: the Dallas Lifespan Brain Study (DLBS) (108 participants aged 40–70 years with 62.9% female participants; Test Set 2, external), the Parkinson’s Progression Markers Initiative (PPMI) (108 healthy controls aged 40 to 70 years with 39.8% female participants; Test Set 3, external), and the Information eXtraction from Images (IXI) cohort (319 participants aged 40–70 years with 59.2% female participants; Test Set 4, external). The demographic characteristics of each dataset are summarized in Table 1 and Fig. 1A, while the study workflow is illustrated in Fig. 1.

### DL predicts demographic properties from 3D brain MRI across multiple cohorts

**Fig. 2:**
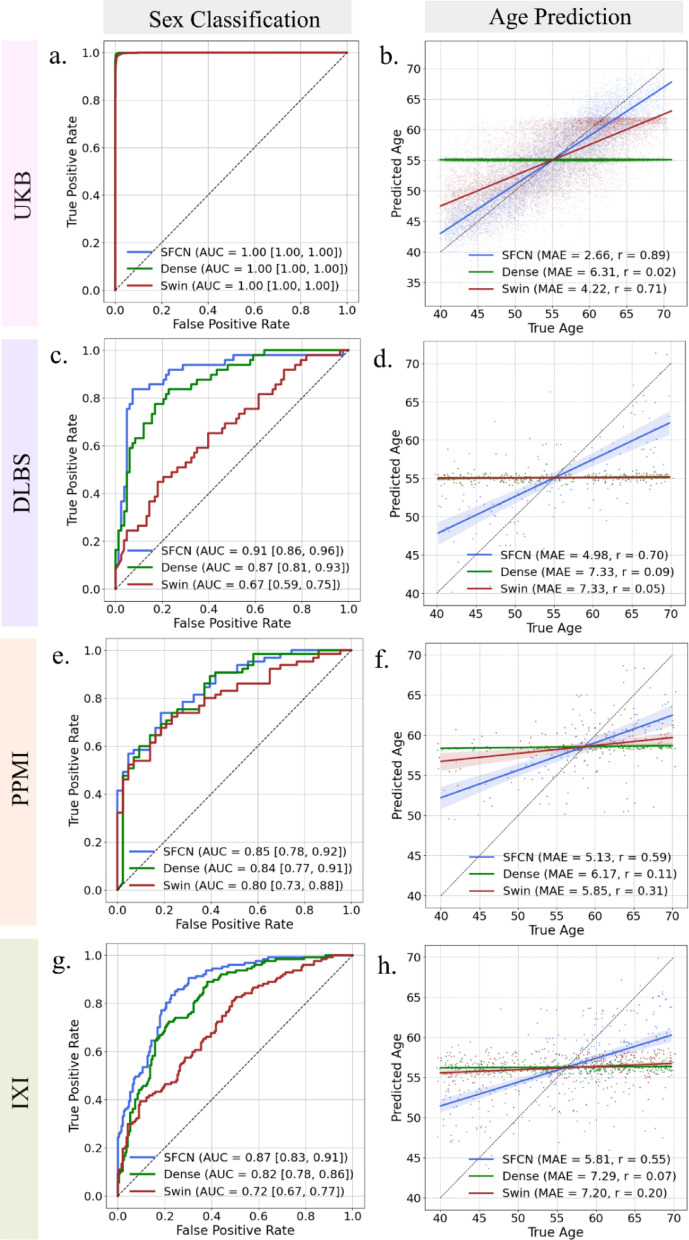
Model comparison. Performance comparison of three deep learning architectures—SFCN (Simple Fully Connected Network, shown in blue), DenseNet (shown in green), and Swin Transformers (shown in red)—evaluated across four neuroimaging datasets. The left column (panels **a**, **c**, **e**, **g**) shows ROC curves for sex classification performance with Area Under Receiver Operating Characteristic Curve (AUC) metrics and confidence intervals, while the right column (panels **b**, **d**, **f**, **h**) displays scatter plots for age prediction accuracy with Mean Absolute Error (MAE) and Pearson’s correlation coefficient (r) metrics. Results are shown for UK Biobank (UKB, panels **a**, **b**), Dallas Lifespan Brain Study (DLBS, panels **c**, **d**), Parkinson's Progression Markers Initiative (PPMI, panels **e**, **f**), and Information eXtraction from Images dataset (IXI, panels **g**, **h**). The diagonal dotted lines in the ROC curves represent random chance performance, while in the scatter plots, they represent perfect prediction

First, we evaluate The Simple Fully Connected Network (SFCN) architecture for predicting sex and age from T1-weighted brain MR imaging studies across the UKB, PPMI, DLBS, and IXI datasets. For sex classification, SCFN achieved an Area Under the Receiver Operating Characteristic Curve (AUC) and 95% confidence interval (CI) of 1.00 [1.00–1.00] in UKB, 0.91 [0.86–0.96] in DLBS, 0.85 [0.78–0.92] in PPMI, and 0.87 [0.83–0.91] in IXI (Fig. 2, left). While the model demonstrated high predictive accuracy, its performance declined in external unseen cohorts compared to the trained UKB cohort.

For age prediction, scatter plots (Fig. 2, right) compare predicted values vs. true values. A strong correlation was observed for the UKB test set, where SFCN achieved a Mean Absolute Error (MAE) of 2.66 years and a Pearson’s r of 0.89. However, performance dropped in the external datasets: DLBS (MAE = 4.98 years, r = 0.70), PPMI (MAE = 5.13 years, r = 0.59), and IXI (MAE = 5.81 years, r = 0.55). Together, these results show that 3D end-to-end deep learning can predict biological variables from brain MRI, albeit with notable performance degradation when applied across external cohorts.

### SFCN outperforms other 3D architectures in sex classification and age prediction

Next, we compared the SFCN model against DenseNet and SwinTransformer for both sex classification and age prediction tasks using isotropic 3D images of size 180*180*180 voxels (Fig. 2, blue, green, red). In UKB, all three models performed equally well for the sex classification, achieving an AUC of 1.00 [1.00–1.00]. However, in DLBS, DenseNet achieved 0.87 [0.81–0.93], while SwinTransformer showed a lower AUC of 0.67 [0.59–0.75]. In PPMI, AUCs were 0.84 [0.77–0.91] for DenseNet and 0.80 [0.73–0.88] for SwinTransformer. To compare the predictive performance of three different models, we conducted pairwise Delong tests with Bonferroni correction to account for multiple models comparisons (α = 0.05/3 ≈ 0.017 for three pairwise comparisons for the three models) and found that the SwinTransformer’s performance was significantly lower than SFCN (adjusted p < 0.017) in all cohorts except PPMI, while DenseNet did not differ significantly from SFCN on external test sets. Detailed description of the performance with Area Under Precision Recall Curves (AUPRCs) are reported in Table 2.

**Table 2:** Performance measures

Cohort	Model	AUC (95% CI)	AUPRC (95% CI)	MAE (95% CI)	Pearson r (95% CI)
UKB	SFCN	0.9997 (0.9996–0.9998)*	0.9996 (0.9995–0.9998)§	**2.66 (2.63–2.69)**	**0.89 (0.89–0.90)**
	Dense	**0.9999 (0.9998–0.9999)**	**0.9998 (0.9998–0.9999)**	6.32 (6.24–6.38)***	0.02 (− 0.00–0.03)
	Swin	0.9993 (0.9991–0.9995)***	0.9992 (0.9990–0.9994)***	4.22 (4.16–4.28)***	0.71 (0.70–0.72)
DLBS	**SFCN**	**0.91 (0.85–0.96)**	**0.86 (0.76–0.94)**	**4.97 (4.35–5.55)**	**0.70 (0.61–0.77)**
	Dense	0.87 (0.80–0.92)§	0.80 (0.70–0.90)§	7.34 (6.52–8.08)***	0.10 (− 0.10–0.30)
	Swin	0.67 (0.58–0.76)***	0.59 (0.45–0.71)***	7.34 (6.57–8.11)***	0.05 (− 0.14–0.24)
PPMI	**SFCN**	**0.85 (0.77–0.92)**	**0.91 (0.85–0.95)**	**5.12 (4.44–5.87)**	**0.58 (0.46–0.69)**
	Dense	0.84 (0.76–0.91)§	0.87 (0.76–0.95)§	6.20 (5.34–7.11)***	0.11 (− 0.01–0.20)
	Swin	0.80 (0.72–0.88)§	0.88 (0.81–0.94)§	5.82 (5.04–6.64)*	0.32 (0.14–0.50)
IXI	**SFCN**	**0.87 (0.83–0.90)**	**0.82 (0.76–0.87)**	**5.80 (5.36–6.29)**	**0.55 (0.45–0.62)**
	Dense	0.82 (0.78–0.86)*	0.73 (0.65–0.80)**	7.29 (6.78–7.80)***	0.07 (− 0.05–0.19)
	Swin	0.72 (0.66–0.77)***	0.66 (0.58–0.74)***	7.16 (6.73–7.64)***	0.20 (0.10–0.30)

The table presents the performance measures of three deep learning models (SFCN, DenseNet, and SwinTransformer) across four datasets (UKB, DLBS, PPMI, and IXI) for sex classification and age prediction tasks. The evaluation metrics include AUC and AUPRC with 95% confidence intervals for sex classification, and MAE and Pearson’s correlation coefficient for the age prediction task. SFCN consistently demonstrates strong performance across all cohorts, particularly in MAE and Pearson’s r for age prediction. Densenet shows competitive AUC and AUPRC in UKB but exhibits lower performance in other datasets. Statistical tests for comparing the models performances within each cohort were done using the DeLong test for AUCs and AUPRCs, and the Wilcoxon signed-rank test for MAE and p-values were evaluated. Bold text denotes the best performing model for each metric and cohort. Statistical significance markers are represented with § for *p* > 0.017, * for *p* < 0.017, ** for *p* < 0.003, and *** for *p* < 0.0003 indicate the degree of underperformance of a model relative to the best-performing model for each metric within a given cohort

For age prediction, in the UKB test set, DenseNet and SwinTransformer performed worse than SFCN, with MAEs of 6.31 years and 4.86, and Pearson's r values of 0.02 and 0.60, respectively. Performance further decreased in external datasets, with DenseNet achieving an MAE of 7.33 years (r = 0.09) in DLBS, 6.17 years (r = 0.11) in PPMI, and 7.29 years (r = 0.07) in IXI. Similarly, SwinTransformer yielded an MAE of 7.35 years (r = 0.03) in DLBS, 5.85 years (r = 0.31) in PPMI, and 7.32 years (r = 0.08) in IXI. To compare the three age prediction models, we conducted pairwise Wilcoxon signed-rank tests on the three model pairs with Bonferroni correction to account for multiple corrections (α = 0.05/3 ≈ 0.017 with three pairwise comparisons for three models). The results confirmed that SFCN significantly outperformed both DenseNet and SwinTransformer in all cohorts (adjusted *p* < 0.017). Together, these results show that SFCN consistently outperforms both Densenet and SwinTransformer architectures across all external cohorts for predicting general clinical and biological properties from 3D brain MRI. Further training characteristics for all models can be found under Supplementary Figure S4.

To further assess the impact of optimization choices, we performed additional experiments with alternative training strategies for the age prediction task (Supplementary Figs. S1–S2). Architecture-specific refinements and learning rate optimization with adaptive scheduling yielded modest improvements for DenseNet and minimal gains for SwinTransformer (Fig. S1). Retraining both models on UKB using MSE loss and a OneCycle learning rate policy further improved performance (Fig. S2); however, the overall performance ranking across architectures remained largely unchanged. These findings suggest that more complex architectures exhibit greater sensitivity to hyperparameter choices and may require more extensive, tailored optimization to reach their full potential.

### Minimal bias in SFCN model performance across age and sex groups

**Fig. 3:**
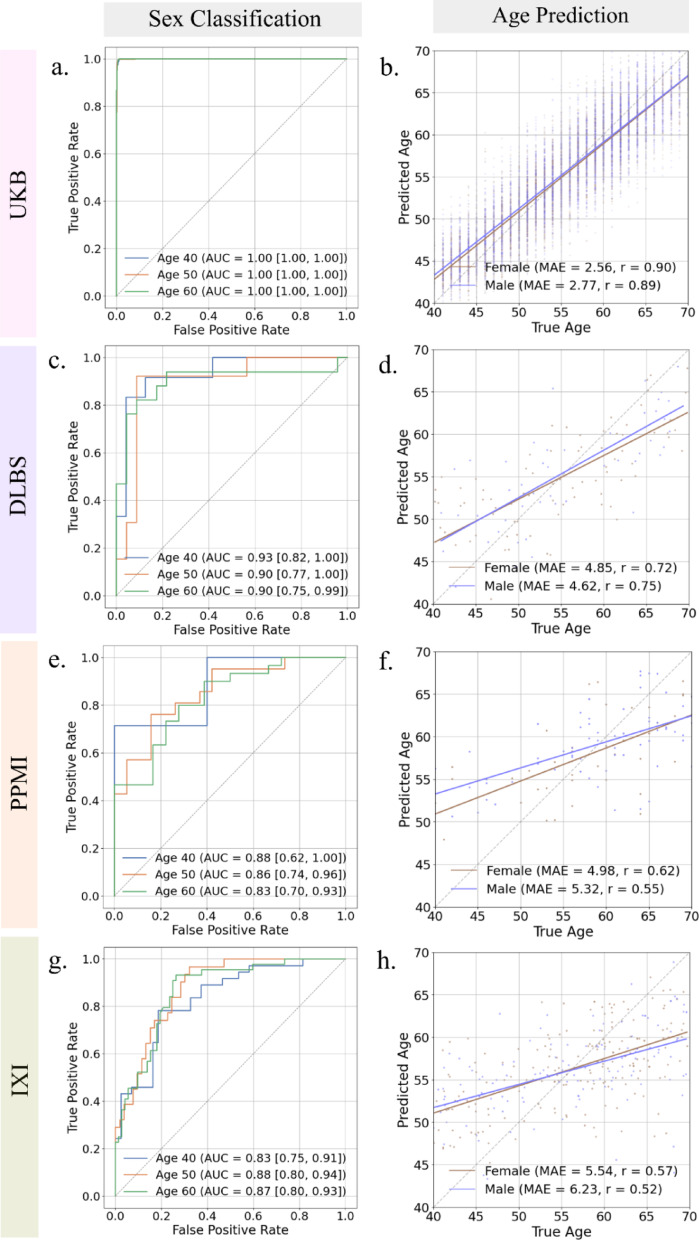
Bias analysis of the SFCN model. Analysis of potential age and sex biases in the SFCN (Simple Fully Connected Network) model across four neuroimaging datasets. The left column (panels **a**, **c**, **e**, **g**) evaluates sex classification performance across three age groups (40–50 years shown in blue, 50–60 years in orange, and 60–70 years in green) using ROC curves with Area Under Receiver Operating Characteristic Curve (AUC) metrics and confidence intervals. The right column (panels **b**, **d**, **f**, **h**) examines age prediction accuracy separately for females (brown) and males (violet), displaying Mean Absolute Error (MAE) and Pearson’s correlation coefficients (r). Results are presented for UK Biobank (UKB, panels **a**, **b**), Dallas Lifespan Brain Study (DLBS, panels **c**, **d**), Parkinson's Progression Markers Initiative (PPMI, panels **e**, **f**), and Information eXtraction from Images dataset (IXI, panels **g**, **h**). Diagonal dotted lines represent random chance performance in ROC curves and perfect prediction in age prediction scatter plots

To assess potential biases in model performance, we evaluated the best-performing model, SFCN, across different age and sex groups. For age bias in sex classification (Fig. 3, left), SFCN maintained a high performance with an AUC of 1.00 [1.00, 1.00] across all age groups in UKB, while in DLBS, AUC values were 0.93 [0.82, 1.00], 0.90 [0.77, 1.00], 0.90 [0.75, 0.99] for age groups of 40–50, 50–60, and 60–70. Similarly, PPMI yielded AUCs of 0.88 [0.62, 1.00] (40–50 years old), 0.86 [0.74, 0.96] (50–60 years old), 0.83 [0.70, 0.93] (60–70 years old), and IXI achieved AUCs of 0.83 [0.75, 0.91] (40 to 50 years old), 0.88 [0.80, 0.94] (50–60 years old), 0.87 [0.80, 0.93] (60–70 years old). These results indicate that age did not introduce significant bias in sex classification across cohorts.

For sex bias in age prediction, we analyzed SFCN performance across male and female groups (Fig. 3, right). In UKB, the model performed slightly better for females (MAE = 2.56 years, r = 0.90) than males (MAE = 2.77 years, r = 0.89). In DLBS, the model was more accurate for males (MAE = 4.62 years, r = 0.75) than females (MAE = 4.85 years, r = 0.72). In PPMI, performance was lower for males (MAE = 5.32 years, r = 0.55) than females (MAE = 4.98 years, r = 0.62), and in IXI, the model again showed lower accuracy for males (MAE = 6.23 years, r = 0.52) than females (MAE = 5.54 years, r = 0.57). These results suggest that age prediction performance was slightly lower for males in most cohorts, though differences were not substantial, thus indicating no sex bias in age prediction.

### DL reveals consistent task-specific attention patterns for discriminative biomarkers

**Fig. 4:**
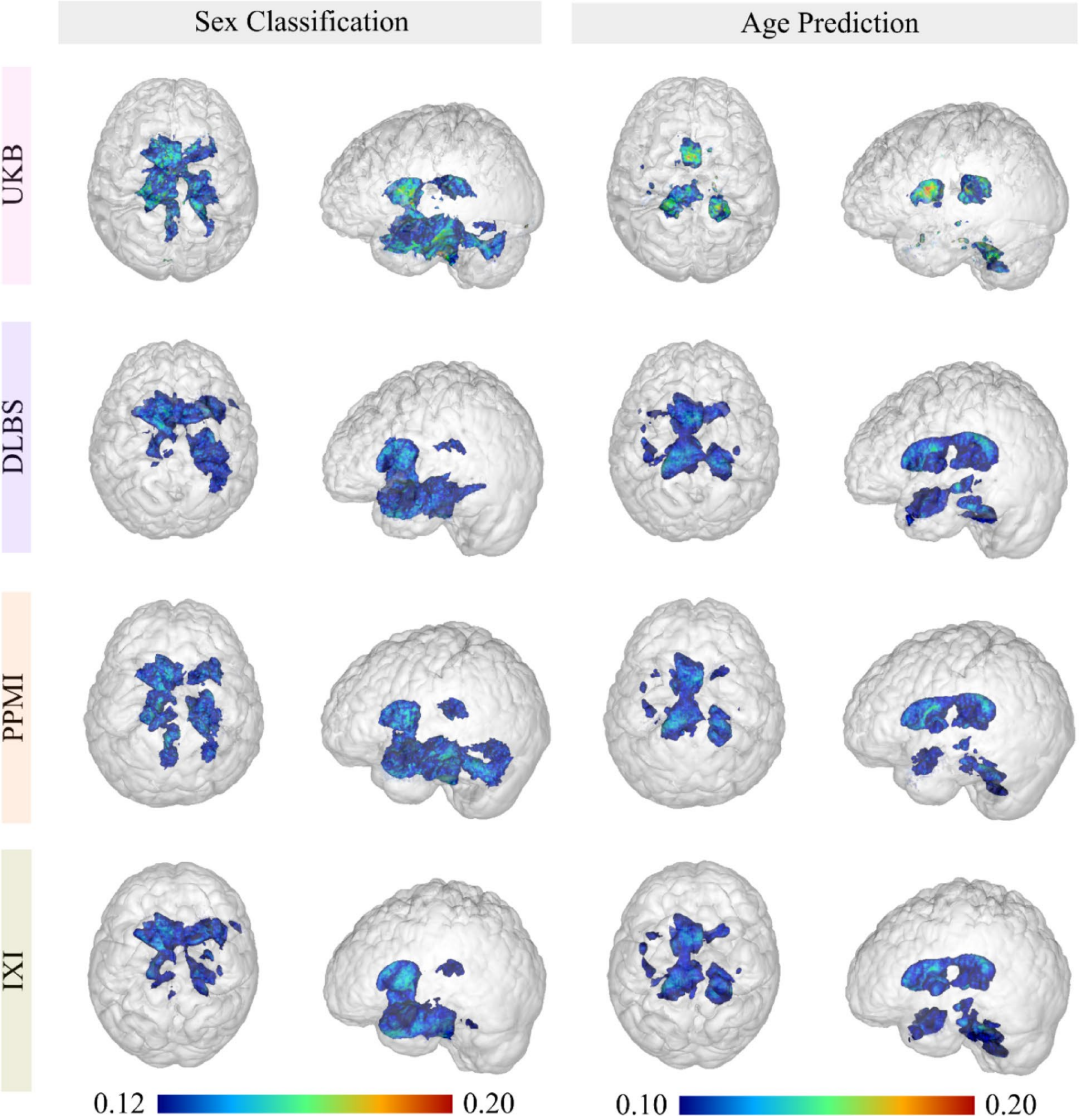
Average heatmaps. Visualization of the SFCN (Simple Fully Connected Network) model’s attention patterns across brain regions using 3D glass brain representations with aggregated heatmap overlays. Each panel presents axial (top-down) and sagittal (left-side) views, where the blue-green–red spectrum represents the intensity of model attention, averaged across all subjects. The left column (panels **a**, **c**, **e**, **g**) depicts attention patterns for sex classification, while the right column (panels **b**, **d**, **f**, **h**) shows patterns for age prediction. Results are displayed for four neuroimaging datasets: UK Biobank (UKB, panels **a**, **b**), Dallas Lifespan Brain Study (DLBS, panels **c**, **d**), Parkinson’s Progression Markers Initiative (PPMI, panels **e**, **f**), and Information eXtraction from Images (IXI, panels **g**, **h**). Attention coefficients were computed using Gradient x Input attribution maps. The values were derived by computing the element-wise product of the input image and its gradients with respect to the predicted class, followed by normalization of the absolute values. The color bars at the bottom indicate the range of these normalized values, spanning from the minimum threshold (0.10–0.12) to the maximum (0.20)

To investigate spatial consistency of model predictions across cohorts, we examined SFCN's attention patterns using gradient-based saliency maps across all four cohorts for both tasks. As illustrated in Fig. 4, the average heatmaps of all subjects in each cohort revealed task-specific attention normalized from 0 to 1 with a threshold relevant for each task. Attention patterns showed notable consistency across cohorts for each task, suggesting that the model may identify generalizable neuroanatomical features rather than dataset-specific artifacts. Upon qualitative analysis, model attention showed to be predominantly distributed across the brainstem, central subcortical structures, and surrounding parts of the limbic lobe, insular cortex, cerebellum, temporal lobe, and occipital lobe for both tasks. While these findings provide insights into the model’s interpretability, further validation is needed to confirm their biological and clinical significance. For a more detailed view of slice-wise attention distribution, see the 2D heatmaps in Supplementary Figures S2 and S3.

### DL enables robust correlation estimates with grey matter region volumes

**Fig. 5:**
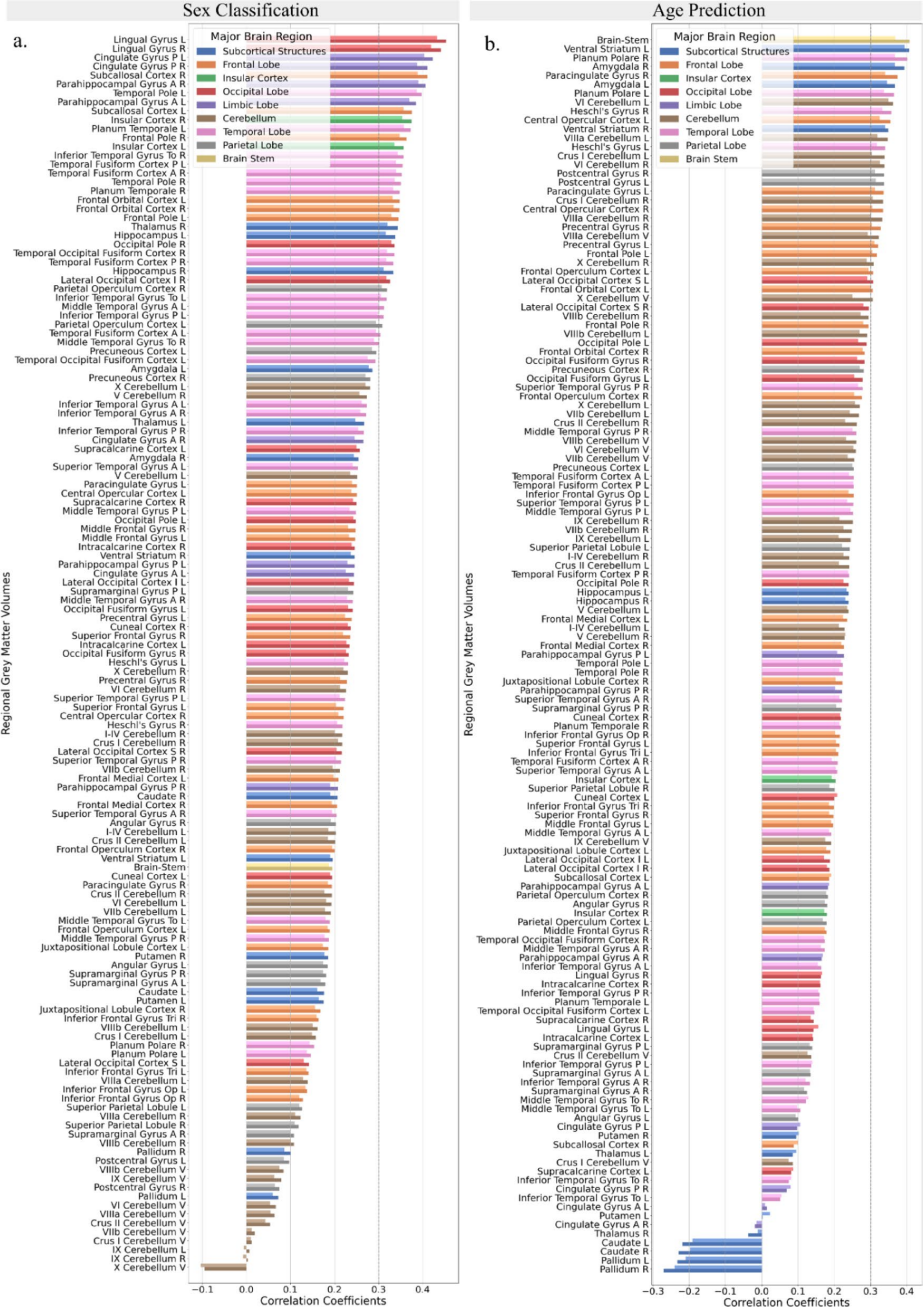
Correlations plots. Correlations between 139 Gy matter region volumes and sex/age characteristics. **a** Sex classification: Pearson’s correlation coefficients of regional brain volumes with ground truth sex labels (lighter shade) and model-predicted scores (darker shade). **b** Age prediction: Pearson’s correlation coefficients of regional volumes with chronological age (lighter shade) and model-predicted age scores (darker shade). Brain regions are grouped into nine major anatomical categories: subcortical structures (blue), frontal lobe (orange), insular cortex (green), occipital lobe (red), limbic lobe (purple), cerebellum (brown), temporal lobe (pink), parietal lobe (gray), and brain-stem (olive). Abbreviations were assigned to hemispheric divisions (L: Left, R: Right), anatomical subdivisions (A: Anterior, P: Posterior, S: Superior, I: Inferior), and specific locations (V: Vermis, To: Temporo-occipital part, Tri: Pars triangularis, Op: Pars opercularis). Correlation coefficients range from − 0.1 to 0.4 for sex classification and − 0.3 to 0.3 for age prediction

Finally, to quantitatively assess the relationship between SFCN’s predictions and underlying brain structures, we analyzed correlations between grey matter region volumes and both prediction scores and ground truth (GT) labels in the UKB test set (Fig. 5). For each region, we calculated Pearson’s correlation coefficients between volumes and prediction scores (r_prediction; darker shade) and then between volumes and ground truth labels (r_label; lighter shade), enabling direct comparison of model-captured versus actual neuroanatomical associations.

In sex classification, the regions demonstrating the strongest volume correlations (> 0.4) with prediction scores were the Lingual Gyrus, the posterior division of the Cingulate Gyrus, and the right anterior division of the Parahippocampal Gyrus. Meanwhile, for age prediction, the Brain Stem, Ventral Striatum, and Planum Polare in the temporal lobe exhibited absolute volume correlations above 0.4, suggesting that these regions play a key role in age-related neuroanatomical changes captured by the model. For both tasks, volume correlations with prediction scores and the GT labels were observed to be consistent across all brain regions, indicating strong alignment with model predictions and anatomical variations.

## Discussion

Predicting biological characteristics such as age and sex from structural brain MRI has been demonstrated with high accuracy through a range of deep learning architectures [37, 45, 65]. While the field has seen significant progress, many studies have recently investigated brain age with a focus on explainability but were lacking in rigorous external validation across independent unseen cohorts [36, 44, 51–53, 66–70]. Conversely, earlier work that evaluated multi-site reliability or external generalizability often provided limited insight into model interpretability [37, 45]. In the present study, we address these complementary gaps by jointly evaluating two key dimensions that are critical for clinical translation: cross-cohort robustness and model interpretability. Specifically, we assessed the generalizability and explainability of three 3D end-to-end architectures—SFCN, DenseNet, and SwinTransformer—across one internal and three external cohorts for age prediction and sex classification. Although all models performed strongly on the internal UK Biobank test set, performance declined when evaluated on unseen external datasets. However, SFCN consistently demonstrated superior generalization, outperforming DenseNet and SwinTransformer across cohorts in both tasks, suggesting that simpler architectures may generalize more reliably to heterogeneous data.

The challenge of model generalization is well recognized in neuroimaging deep learning. Many prior studies evaluated performance using hold-out subsets from the same cohort [36, 44, 51–53, 66–70]. However, without external validation, it remains difficult to assess whether these models generalize reliably beyond the specific population and acquisition conditions on which they were trained. The inclusion of three independent external cohorts in this study provides a more realistic test of model robustness across heterogeneous populations and imaging settings, consistent with recent studies advocating for stronger validation frameworks [41, 45, 68, 71, 72]. In addition, training on a large population-based dataset such as UK Biobank may help models learn representations that are more robust to demographic and technical variability, potentially improving their transferability across cohorts.

The gradient-based saliency analysis revealed that attention patterns were notably consistent across cohorts for each task, with predominant focus on brainstem, subcortical structures, and limbic and insular regions for both age prediction and sex classification. This cross-cohort consistency suggests that the model may identify generalizable neuroanatomical features rather than dataset-specific artifacts. To provide a quantitative basis for interpreting these saliency patterns, we examined the relevance of regional gray matter volumes for each task by correlating ground-truth labels (age/sex) and model predictions with regional volumes. This analysis establishes which brain regions show structural associations with the predicted outcomes, offering a reference for evaluating whether the saliency maps highlight neuroanatomically meaningful regions. Many of the regions highlighted by the saliency maps have been previously associated with age-related changes and sex differences [73, 74], supporting the model’s potential to capture meaningful structural variations. Recent studies have employed different explainability methods to interpret the biological emphasis of the model [66, 68, 69, 75, 76]. Our goal was to assess explainability in a comparative framework across different tasks, and cohorts.

In addition to model generalization, our analysis examined potential **biases** in model performance across demographic subgroups. Many studies have previously highlighted the influence of age on sex classification performance and the role of sex differences in age prediction [64, 77–80]. Our results further emphasize the need for robust validation frameworks that include diverse and independent datasets to ensure broad model applicability across different populations.

While this study provides valuable insights into the evaluation of 3D DL architectures for neuroimaging, it is not without its limitations. First, the training cohort, while large, was limited in age range and population diversity. Future research should incorporate datasets spanning wider age ranges and diverse ethnic populations to validate the model's generalizability across more diverse demographic subgroups.

Second, while SFCN demonstrated superior performance compared to the tested architectures under the training configurations explored in this study, the comparison was not based on fully architecture-specific hyperparameter optimization and may not fully capture optimal performance for DenseNet and Swin Transformer. Specifically, Transformer-based architectures may require substantial domain-specific optimization for 3D medical imaging. Future work should include a greater number of 3D-DL models alongside extensive, architecture-tailored hyperparameter tuning to more comprehensively benchmark the relative potential of these models for 3D neuroimaging. [71, 74].

Third, while gradient-based saliency maps may provide general insights into regional contributions to model predictions, the interpretation of these explainability results should be approached with caution. Gradient-based attribution methods have known theoretical and empirical limitations [81–85]. Validating the reliability of such methods is essential before drawing neurobiological conclusions. To provide a quantitative basis for visual inspection of the saliency maps, we examined the relevance of regional gray matter volumes for each task by correlating ground-truth labels and model predictions with regional volumes, pointing at structural associations with the predicted outcomes. Future work could incorporate additional structural features—such as white matter integrity, cortical thickness and surface area.

Despite current limitations, we see a great opportunity to use this model as a basis for applications in degenerative neurological diseases such as Alzheimer’s disease, Frontotemporal Dementia, Parkinson’s disease or Multiple Sclerosis. There is a need to investigate how neurodegeneration affects the results of age and sex predictions, as these factors may differ in affected populations compared to healthy individuals. Understanding these relationships will help refine the accuracy and applicability of the model in clinical settings. In a subsequent phase, we envision significant potential for the model to predict disability scores or milestones of disease worsening, which serve as a critical measure of disease severity. By integrating such predictions, clinicians could gain valuable insights into the progression of disease-related neurodegeneration in diverse disease courses, better reflecting real world data and ultimately improving individual patient management and treatment strategies.

## Conclusion

In conclusion, this study provides a multi-cohort evaluation of 3D deep learning architectures for age and sex prediction from brain MRI, with an emphasis on generalizability and interpretability across cohorts. Across experiments, SFCN demonstrates a favorable balance between predictive performance and computational efficiency, supporting its suitability for large-scale neuroimaging analyses. The study however had its limitations. The training cohort spans a restricted age range, which may limit model performance outside this distribution. While consistent hyperparameter settings were used to ensure fair comparison, exhaustive architecture-specific optimization was not feasible but may affect absolute performance estimates. Additionally, gradient-based explainability methods require more robust quantitative validation to establish their reliability before neurobiological interpretation. Despite these constraints, the present work provides practical insights into the importance of external evaluation frameworks along with interpretability for robust brain MRI–based deep learning models.

## Supplementary Information

Supplementary Material 1.

## Data Availability

This study utilized MRI data from the UK Biobank under Application Number 92261. Researchers can request access to the UK Biobank data through the official application process (https://www.ukbiobank.ac.uk/enable-your-research/apply-for-access). External validation was conducted using data from the Dallas Lifespan Brain Study (DLBS) that can be accessed via (https://fcon_1000.projects.nitrc.org/indi/retro/dlbs.html) or (https://openneuro.org/datasets/ds004856/versions/1.0.0), Parkinson’s Progression Markers Initiative (PPMI) that can be accessed via (https://www.ppmi-info.org), and Information eXtraction from Images (IXI), all of which can be accessed via (https://brain-development.org/ixi-dataset).

## Code availability

The underlying code for this study is available on Github and can be accessed via this link https://github.com/jrad9921/RadBrainDL. Codebase is continuously developed and might evolve after publication.
