# Supplementary material for: Generalizable and explainable deep learning for brain MRI: a multi-cohort evaluation of 3D architectures for age and sex prediction
Source: Brain Inform. 2026 Jul 6;13(1):31. doi: 10.1186/s40708-026-00316-y (PMC13342005; doi:10.1186/s40708-026-00316-y)
Supplement: Supplementary file 1 — Supplementary Material 1. [file 40708_2026_316_MOESM1_ESM.docx]

### **Supplementary**

#### **Table S1. Dataset Information**

| **Dataset** | **Full cohort name** | **MRI Type** | **MRI Sequence** | **Source** |
| --- | --- | --- | --- | --- |
| UKB | UK Biobank | T1w | MPRAGE | <https://biobank.ctsu.ox.ac.uk/crystal/crystal/docs/brain_mri.pdf> |
| DLBS | Dallas Lifespan Brain Study | T1w | MPRAGE | <https://fcon_1000.projects.nitrc.org/indi/retro/dlbs.html> |
| PPMI | Parkinson's Progression Markers Initiative | T1w | MPRAGE | <https://www.ppmi-info.org/> |
| IXI | Information eXtraction from Images | T1w | n.a. | <https://brain-development.org/ixi-dataset/> |

Links for finding details about image acquisition protocol followed by each cohort.

####

#### **Table S2. Parameter Comparison**

| **Task** | **Model** | **Parameters** | **GPU memory (MiB)** | **Training Time (seconds)** |
| --- | --- | --- | --- | --- |
| Age Prediction | SFCN | 2950401 | 13,308 | 206980.4787 |
|  | Densenet121 | 11243649 | 13,068 | 62740.61855 |
|  | SwinTransformer | 34199239 | 34,570 | 301686.4642 |
| Sex Classification | SFCN | 2950466 | 13,308 | 156551.9253 |
|  | Densenet121 | 11244674 | 13,068 | 178049.7876 |
|  | SwinTransformer | 34199336 | 34,570 | 205635.2252 |

Table displaying the comparison of the compute power consumed by each model.

####

#### **Fig S1. Hyperparameter tuning using smaller but age-diverse datasets.**


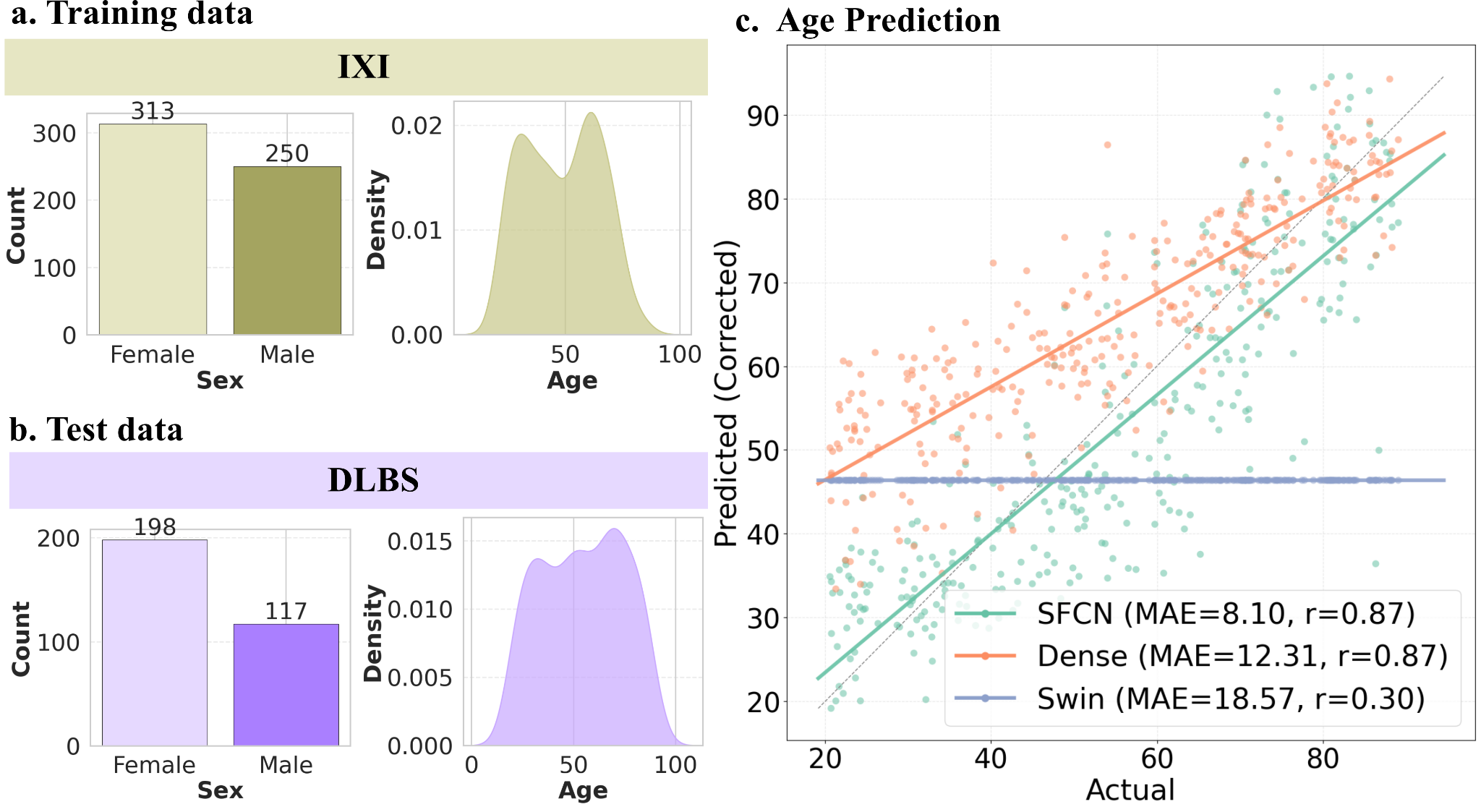


Hyperparameter tuning using (a) IXI dataset for training: Sex (Female/Male: 313/250) and age (20-90 years) distribution (b) DLBS dataset for testing: Sex (Female/Male: 198/117) and age (20-90 years) distribution (c) Scatter plots of predicted (and corrected) age versus chronological age on the DLBS test set for the same models trained on IXI. Predictions are shown after bias correction. Solid lines indicate linear fits and the dashed diagonal represents perfect agreement. Mean absolute error (MAE) and Pearson’s correlation coefficient (r) are reported for each architecture. The results are with training following a learning rate finder.

**Fig S2: Hyperparameter optimization for age prediction with Densenet121 and SwinTransfomer**


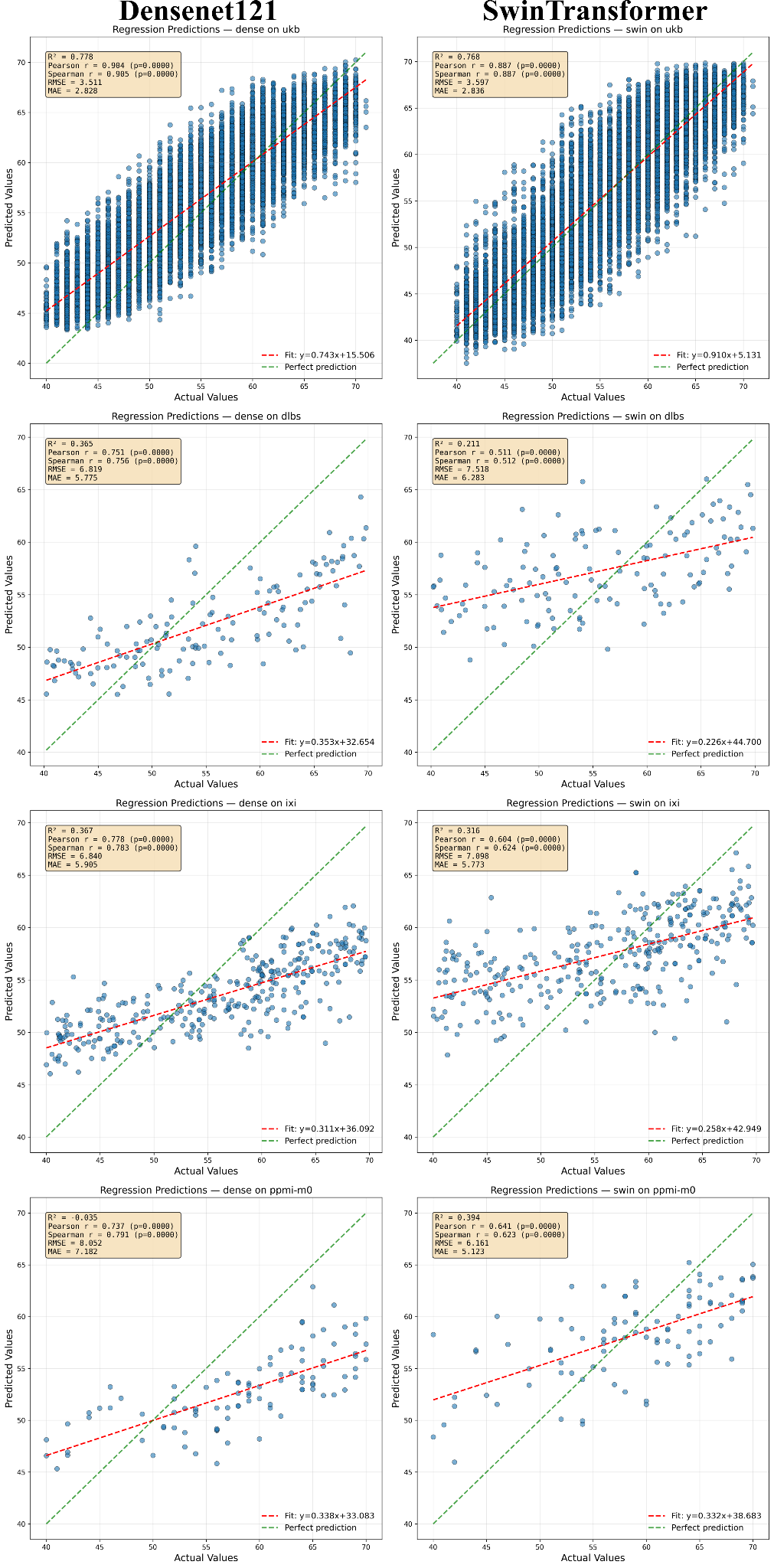


Hyperparameter optimization for DenseNet-121 and Swin Transformer for brain age prediction, tested across multiple cohorts (UKB, DLBS, IXI, and PPMI) using a training configuration adopted from Siegel et al [^69^](https://paperpile.com/c/fO1yEF/nLDGc). Both models were trained with Mean Squared Error (MSE) loss this time, and a OneCycle learning rate scheduler with maximum learning rate of 1e-2 for Densenet121 and 1e-4 for SwinTransformer. Scatter plots show predicted versus chronological age, with the red dashed line indicating the fitted regression and the green dashed line representing perfect prediction. Performance metrics (R², Pearson’s r, Spearman’s ρ, RMSE, and MAE) are reported for each cohort.

#### **Fig S2. 2D Heatmaps for sex classification**


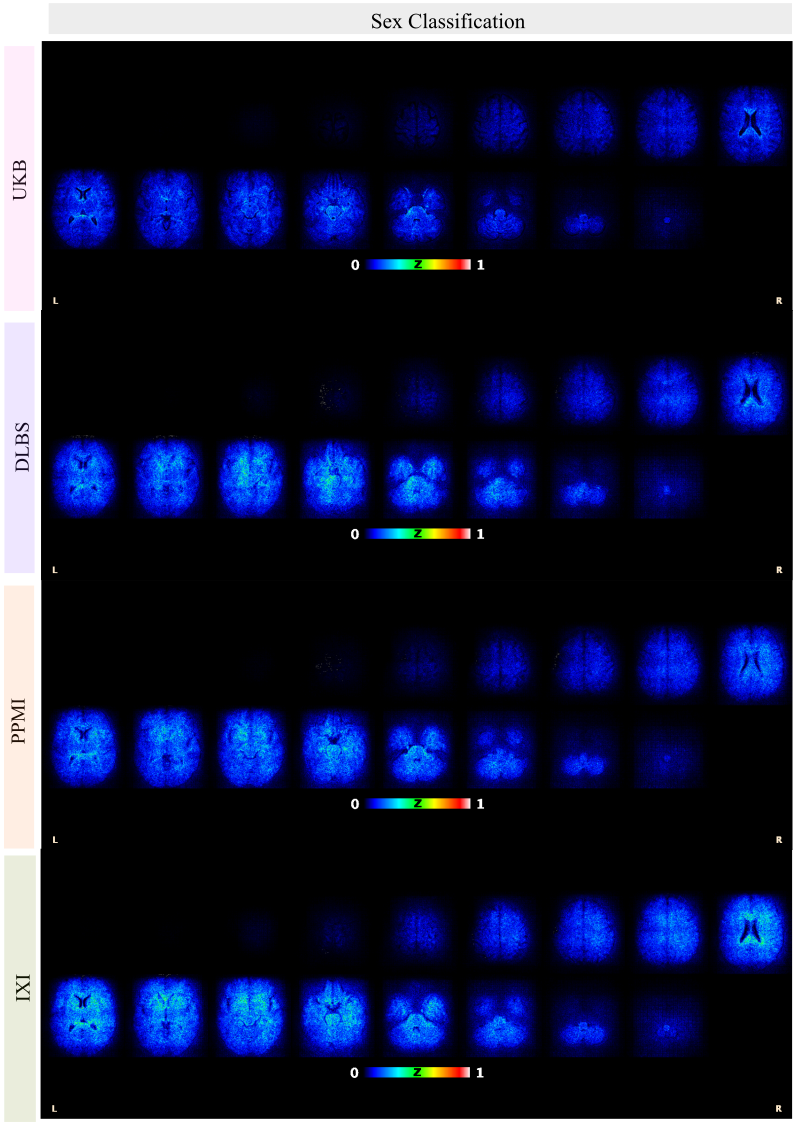


Axial brain slices in gaps of 10, demonstrating sex classification patterns for the Simple Fully Connected Network (SFCN) in four neuroimaging datasets (UKB, DLBS, PPMI, and IXI). Eighteen axial slices from top to bottom of the brain are represented in two rows of nine slices for each dataset. Left (L) and right (R) orientation markers are provided for each row, and a standardized colorbar appears beneath each dataset's slices. The intensity values are displayed on a scale of 0 to 1, represented by a blue-green-red colormap, with predominant activations appearing in blue.

#### **Fig S3. 2D Heatmaps for age prediction**


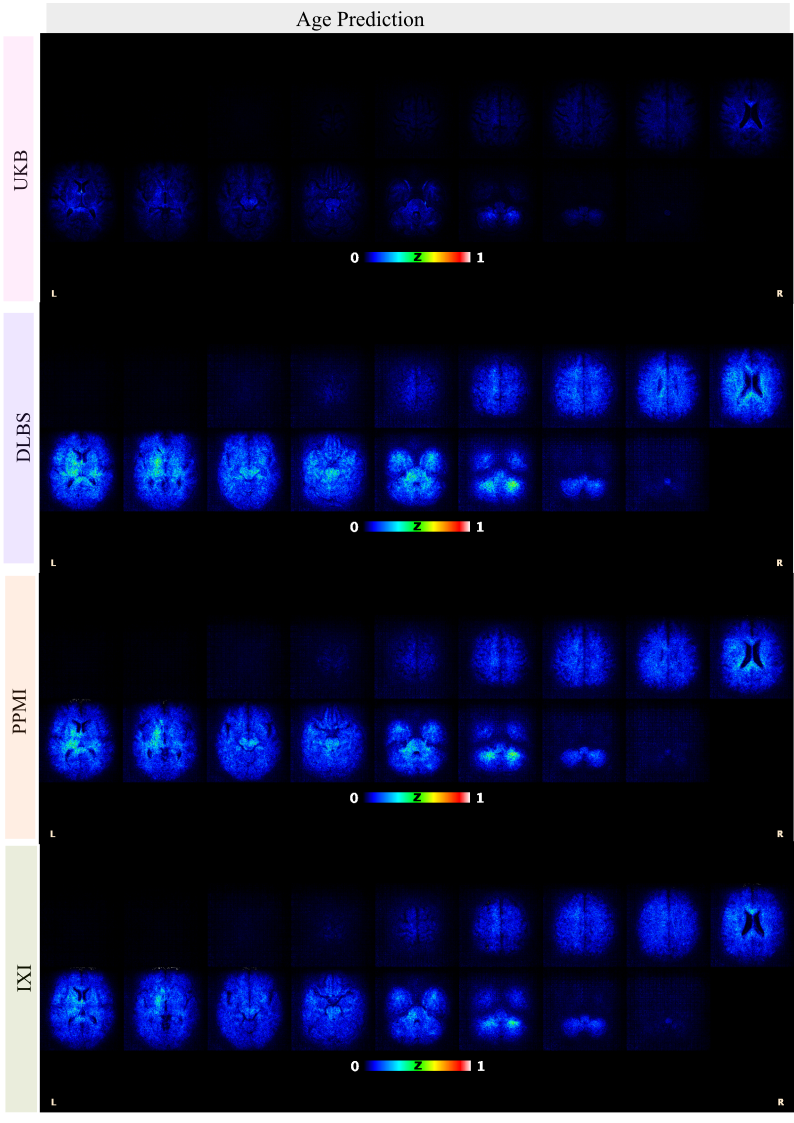


Axial brain slices in gaps of 10, demonstrating age prediction patterns for the Simple Fully Connected Network (SFCN) in four neuroimaging datasets (UKB, DLBS, PPMI, and IXI). Eighteen axial slices progressing from top to bottom of the brain are represented in two rows of nine slices for each dataset. Left (L) and right (R) orientation markers are provided for each row, and a standardized colorbar appears beneath each dataset's slices. The intensity values are displayed on a scale of 0 to 1, represented by a blue-green-red colormap, with predominant activations appearing in blue.

#### **Fig S4. Training and Validation Losses for each model across both cohorts**


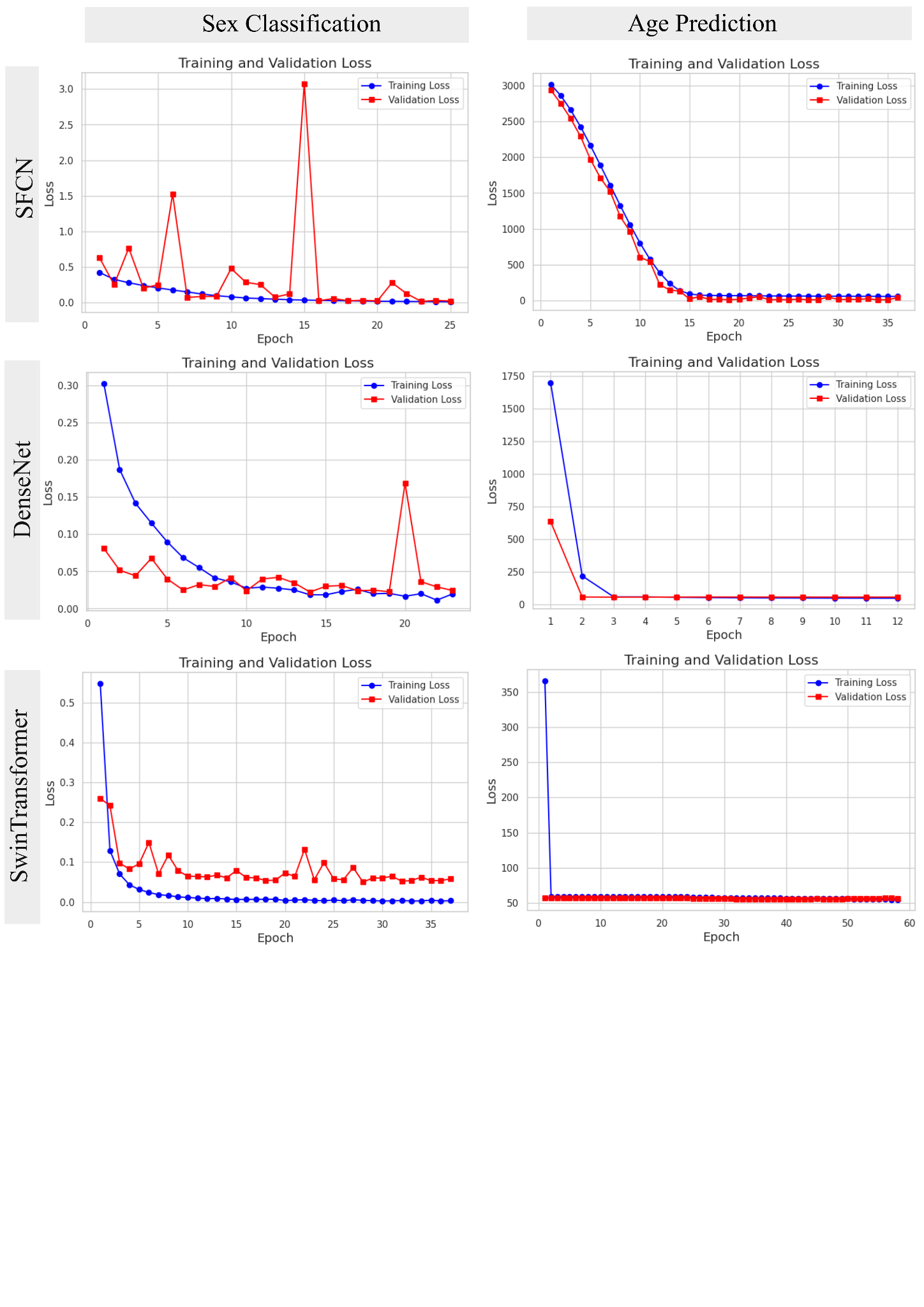


Training and Validation losses visualized for each model for all epochs until the epoch with last best loss. Early stopping was applied when the validation loss did not decrease for 10 consecutive epochs.
